# Typical and atypical presentations of myocardial infarction: Symptoms and associated risk factors

**DOI:** 10.5339/qmj.2025.41

**Published:** 2025-06-09

**Authors:** Lina Rasmi AIMbaidin, Raja’ Qasaimeh, Rou’eyh Qasaimeh, Hayat Al-Ali, Mohammad AL-Abidalrazag, Saba Atieh, Ashraf A. Zaghloul

**Affiliations:** 1 Preventive and Community Medicine Department, Hamad Medical Corporation, Doha, Qatar; 2 Department of Obstetrics and Gynecology, Princess Badea Hospital, Irbid, Jordan; 3 Faculty of Medicine, Hashemite University, Zarqa, Jordan; 4 Universiti Sains Malaysia, School of Health Sciences, Kubang Kerian, Kota Bharu, Kelantan, Malaysia.; 5 Emergency Medicine Hospital, Albasheer Hospitals, Amman, Jordan; 6 Department of Medical Laboratory Science, Al-Ahliyya Amman University, Amman, Jordan; 7 High Institute of Public Health Alexandria University, Alexandria, Egypt *Email: lalmbaidin@hamad.qa

**Keywords:** Acute myocardial infarction (AMI), typical, atypical, delayed diagnosis, Jordan

## Abstract

**Background/Introduction:**

Acute myocardial infarction (AMI) affects around 3 million people annually worldwide. Typical symptoms prompt timely care, while atypical symptoms may delay diagnosis and treatment, increasing the risks of heart failure and sudden cardiac death.

**Objective/Purpose:**

This study aimed to compare typical and atypical myocardial infarction (MI) presentations to enhance early diagnosis, treatment, and prognosis by examining clinical characteristics, chronic comorbidities, history of coronary artery disease, and smoking status at a tertiary hospital in Amman.

**Methods:**

This purposive, descriptive, retrospective study was conducted at the coronary care unit of Al Basheer Hospital in Amman, Jordan, from July 1, 2022, to July 1, 2023. Records of patients diagnosed with MI (ICD-10 code I21) were extracted. Data were analyzed using the IBM SPSS Statistics software (version 25.0). Tests of association were adjusted at the 5% significance level.

**Results:**

The study included 267 MI cases: 60.3% were typical, and 39.7% were atypical. No significant differences were found among age (*p* = 0.58) and sex (*p* = 1.27). Atypical MI was linked to a history of coronary artery disease (*p* < 0.05) and higher diabetes prevalence (*p* < 0.05). Recurrence rates (*p* = 0.41) and artery involvement, especially the left anterior descending artery, were similar between groups. Echocardiography showed identical rates of left ventricular hypertrophy in both types, with tricuspid regurgitation more common in atypical MI.

**Conclusion:**

The study found that atypical MI was linked to higher rates of diabetes and coronary artery disease history compared to typical MI. Recurrence rates, artery involvement, and echocardiography results were similar between both types.

## Introduction

Acute myocardial infarction (AMI) is a life-threatening condition within the spectrum of coronary artery diseases (CAD).^1^ Primary complications of AMI include new-onset mitral regurgitation, left ventricular aneurysm, and ventricular septal rupture, which can impair both diastolic and systolic function, increasing the risk of fatal arrhythmias.^2^ Another severe consequence of AMI is cardiogenic shock (CS), which often arises from a sudden disruption of an atheromatous plaque that exposes subendothelial collagen and necrotic materials, leading to thrombus formation and rapid myocardial ischemia. This ischemia results in ischemic myocyte necrosis, causing a significant reduction in stroke volume, cardiac output, and blood pressure. These changes trigger compensatory mechanisms such as tachycardia and neurohormonal activation; however, they may exacerbate the ischemic state by increasing myocardial oxygen demand. The deterioration of myocardial function leads to a vicious cycle of worsening hemodynamics and systemic effects, including hypotension and renal dysfunction, which can rapidly progress to multiorgan failure. Furthermore, metabolic acidosis and systemic inflammation complicate the clinical picture, heightening the risk of hemometabolic shock, which is associated with significantly higher mortality. In this case, AMI early intervention is essential to break the cycle.^3^ Data indicate that at least one-third of patients die before reaching the hospital, and an additional 40–50% do not survive upon arrival. Meanwhile, patients who receive early perfusion treatment, such as thrombolytic therapy within 30 minutes of arrival or percutaneous coronary intervention within 90 minutes, tend to experience better outcomes.^2^ This underscores the importance of timely medical intervention in AMI, as it is critical for improving patient outcomes and preventing severe complications, including fatal arrhythmias and AMI-induced CS. Chest, arm, or jaw pain that feels dull, heavy, tight, or crushing is typical of myocardial infarction (MI) symptoms. In contrast, atypical symptoms often involve epigastric or back pain or a burning, stabbing sensation similar to indigestion.^4^ Atypical symptoms cannot be easily diagnosed since there is a lack of knowledge about them and their recognition in the world’s standard population. This further reduces the chance of having an emergency dispatch with the increased risk of 30-day mortality compared to patients with a typical presentation.^5^ One of the atypical manifestations of AMI is silent MI, which independently increases the risk of sudden cardiac death and is also associated with a higher risk of heart failure.^6,7^ The most favorable prognosis is observed in MI patients who receive early and successful reperfusion and have preserved left ventricular function.^2^ All of the points discussed above emphasize the necessity of studying the risk factors associated with atypical MI and understanding the differences in presentation and risk factors between typical and atypical presentations. This enhances diagnostic accuracy and reduces the time required for diagnosis, resulting in a better overall prognosis. Many authors have concluded that the risk factors for atypical MI include advanced age (65 years and older), female gender, and prevalent medical comorbidities such as diabetes mellitus and hypertension.^8–14^ This descriptive study aims to characterize the percentage of patients presenting with atypical AMI and to examine differences in symptoms and associated factors, such as age, gender, and comorbidities, compared to typical MI. Additionally, the study explores variations between typical and atypical MI cases in terms of recurrence rates, artery involvement, and echocardiography findings. The results will provide valuable insights into the different presentations of the disease, helping doctors avoid future misdiagnoses and improve patient care.

## Methods

### Study design

A descriptive, retrospective, cross-sectional study was conducted, extracting data from the Hakeem system files at Al Basheer Hospital, Amman, Jordan. The population under study was divided according to the primary outcome: typical/atypical MI.

### Study setting

The study occurred at the coronary care unit of Al Basheer Hospital, a 700-bed tertiary hospital with an emergency unit of 90 beds and a critical unit of 26 beds, between July 1, 2022, and July 1, 2023.

### Study population

Records of all patients admitted to the coronary care unit (CCU) at Al Basheer Hospital from July 1, 2022, to July 1, 2023, with a confirmed diagnosis of MI (ICD-10 code I21) were included. All cases were coded and verified by consultants at the Al Basheer Hospital ICU. Thus, we ensured that all cases met the diagnostic criteria for MI.

### Exclusion criteria

Files with more than 50% missing data were excluded from the analysis.

### Study instrument

The data collection survey consisted of two sections:

**Section 1: Patient Identification:** Electronic patients’ records were screened for the ICD-10 code I21 for MI, which is used in the CCU. An MI was diagnosed when at least two of the following criteria were met, as outlined in the Fourth Universal Definition of MI (2018) by the ESC/ACC/AHA/WHF: (symptoms of myocardial ischemia (retrosternal chest pain), new ischemic ECG changes, development of pathological Q waves, imaging evidence of new loss of viable myocardium or new regional wall motion abnormality in a pattern consistent with an ischemic etiology, identification of a coronary thrombus by angiography or autopsy (not applicable for type 2 or 3 MIs).

This section also included variables for identification based on **sex** (male = 0, female = 1) and **age**, classified (35–64 years = 1, 65–74 years = 2, and ≥75 years = 3). Additionally, it documented whether patients presented with **typical symptoms** (chest pain, discomfort, heaviness, or pressure = 1) or **atypical symptoms**. Those with atypical presentations were also recorded, including the type of recovered symptoms: (indigestion = 2, epigastric or abdominal pain = 3, shortness of breath = 4, back or jaw tightness/pain = 5, nausea = 6, vomiting = 7, diarrhea = 8, and generalized feelings of unwellness = 9).

**Section 2: Study Variables:** This survey included the following variables:

**• Medical history:** (the history of the following conditions was recorded, along with their respective ICD codes: CAD = I25.9, diabetes = E11, hypertension = I10, dyslipidemia = E78.5, and other relevant conditions).

**• Smoking status:** (smoker = 0, nonsmoker = 1).

**• Recurrence rate:** (with recurrent MI <9 0 days = 1, without recurrent MI = 2).

**• Artery involvement according to cardiac catheterization results:** (left main coronary artery (LMCA) = 1, right coronary artery (RCA) = 2, left anterior descending artery (LAD) = 3, left circumflex artery = 4, diagonal branches (D1, D2) = 5, septal branches = 6, marginal branches (M1, M2) = 7, acute marginal branch = 8, AV node branch = 9, posterior descending artery = 10).

**• Echocardiography results:** (severe LV dysfunction = 1, LV hypertrophy = 2, mitral regurgitation = 3, tricuspid regurgitation = 4, pulmonary hypertension = 5).

### Statistical analysis

Collected data were analyzed using the IBM SPSS Statistics software (version 25.0). Data were presented in tabular form with frequency and percentages of the variables under study. The statistical test of association (Chi-square test) was used whenever appropriate. The statistical test was adjusted at the 5% significance level throughout the results.

## Ethical Considerations

This research received ethical approval from the Jordanian Ministry of Health’s Research Ethical Committee under reference number MOH/REC/2023/482. All ethical guidelines were followed, ensuring confidentiality and protection of patient data throughout the study.

## Results

Table 1 presents the distribution of the study sample by typical and atypical MI presentation. Of the 267 total MI cases, 60.3% (*n* = 161) were typical, and 39.7% (*n* = 106) were atypical.

### Age group

The age group distribution showed that 64-year-old or older patients accounted for the majority of MI cases in typical and atypical presentations: 75.7% (*n* = 202), with 75.8% (*n* = 122) of typical MI and 75.5% (*n* = 80) of atypical MI.

The least represented age group in both categories was 75+ years, 4.9% (*n* = 13), accounting for 5.6% (*n* = 9) of typical MI and 3.8% (*n* = 4) of atypical MI. There was no significant association between age groups and typical and atypical MI (*X*^2^ = 0.58).

### Sex

Males were predominant in both MI presentations at 77.2% (*n* = 206), constituting 79.5% (*n* = 128) of typical MI cases and 73.6% (*n* = 78) of atypical MI cases. In the total sample of 267 individuals, 22.8% (*n* = 61) were female patients, 20.5% (*n* = 33) of typical MI, and 26.4% (*n* = 28) of atypical MI, with no significant association between the groups based on sex (*X*^2^ *p* = 1.27).

### History of CAD

A previous history of coronary artery disease was present in 29.2% (*n* = 78) of all MI cases, 23.0% (*n* = 37) of typical MI cases, and 38.7% (*n* = 41) of atypical MI cases. Conversely, 77.0% (*n* = 124) of typical MI cases and 61.3% (*n* = 65) of atypical MI cases had no prior history of coronary artery disease. A statistically significant association was detected as regards patients with a positive CAD history and atypical presentation of MI (*X*^2^ = 7.62, *p* < 0.05).

### Diabetes

53.6% (*n* = 143) of the 267 patients had diabetes. The prevalence of diabetes was higher in the atypical MI group, at 66.0% (*n* = 70), than in the typical MI group, at 45.3% (*n* = 73). Normal blood glucose levels were more common in the typical MI group, 54.7% (*n* = 88), than in the atypical MI group, 34.0% (*n* = 36). The association was statistically significant (*X*^2^ = 11.1, *p* < 0.05), showing that the percentage of diabetes was significantly higher in atypical MI.

### Hypertension

65.9% (*n* = 176) of the cases were hypertensive. Hypertension was prevalent in typical and atypical MI groups, with 64.0% (*n* = 103) and 68.9% (*n* = 73), respectively (*X*^2^ = 0.68, *p* > 0.05).

### Hyperlipidemia

The absence of hyperlipidemia was noted in most typical 80.7% (*n* = 130) and atypical 77.4% (*n* = 82) MI cases. Hyperlipidemia was relatively low, with 19.3% (*n* = 31) in the typical MI group and 22.6% (*n* = 24) in the atypical MI group (*X*^2^ = 0.44, *p* > 0.05).

### Smoking

Out of the total sample of 267 cases, 53.9% (*n* = 144) were smokers. Positive smoking history was present in the typical MI cases, with 61.5% (*n* = 99) being smokers, while in the atypical MI group, 57.5% (*n* = 61) were nonsmokers. No significant association was detected.

### Atypical presentations of MI

**Figure 1:**
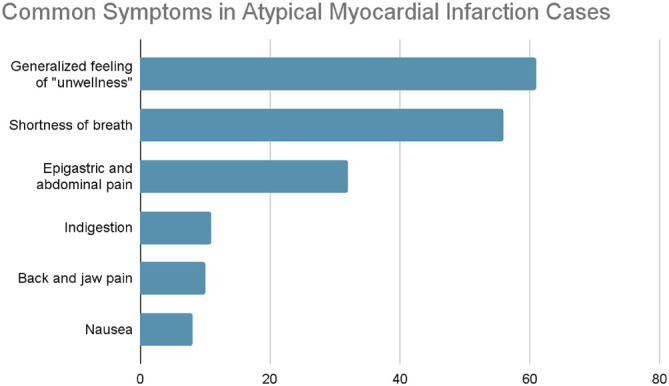
Distribution of common symptoms in atypical MI cases (Amman, 2024). Figure 1 shows the symptom distribution among patients with atypical MI, comprising 39.7% (*n* = 106) of the cases. The most commonly reported symptoms were generalized unwellness, shortness of breath, and epigastric or abdominal pain.

Figure 1 presents the symptoms presented by patients with atypical MI. 39.7% (*n* = 106) of the cases seen in the study have been defined as atypical. Generalized feelings of “unwellness,” shortness of breath, and epigastric or abdominal pain were the most common atypical presenting symptoms. Followed by indigestion, back and jaw pain, nausea, vomiting, and diarrhea. Other symptoms were reported but less frequently, such as headache, dizziness, muscle spasms, and cough.

### Recurrence rate differences between typical and atypical MI

Table 2 shows the distribution of typical and atypical MI recurrence rates.

The percentage of recurrent MI in the typical MI group was 4.3% (*n* = 7) in the typical MI group and 5.7% (*n* = 6) in the atypical MI group (*X*^2^ = 0.41, *p* > 0.05).

Regarding the timing of recent MI among those who experienced recurrence, the highest representation was for MI occurring in the subsequent 3 months in both groups: 94.1% for typical MI and 79.2% for atypical MI. MIs occurring in the subsequent 1–2 months were less common, reported in 5.9% of the typical MI group and 20.8% of the atypical MI group (*X*^2^ = 2.12, *p* > 0.05).

### Artery involvement in patients with typical and atypical MI

**Figure 2:**
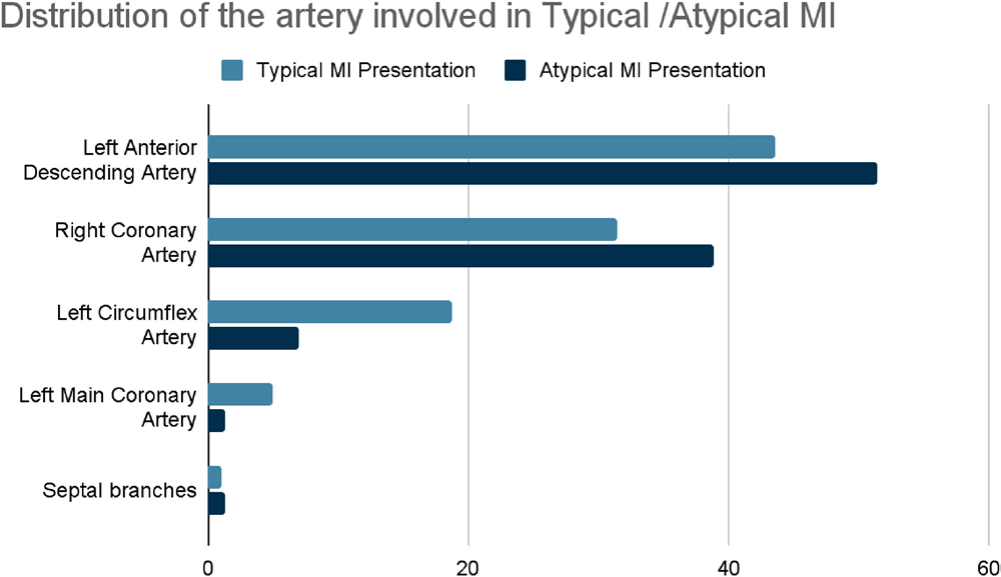
Distribution of the artery involved in typical/atypical MI in the study sample (Amman, 2024). The left anterior ascending artery was the most involved in both groups (typical MI: 43.65%, atypical MI: 51.39%). The lowest involvement in the typical MI group was in the septal branches (1.1%), while in the atypical MI group, it was in the LMCA and septal branches (1.39%).

Figure 2 illustrates the distribution of artery involvement in patients with typical and atypical MI in the study sample. The LAD artery was the most commonly involved in both groups, with 43.65% of typical MI cases and 51.39% of atypical MI cases. Conversely, the septal branches were the least commonly involved in the typical MI group (1.1%). The LMCA and the septal branches had the lowest involvement in the atypical MI group (1.39%).

The study indicates that while the LAD artery is the most frequently involved in typical and atypical MIs, atypical MIs show a slightly higher percentage of LAD involvement. The septal branches and the LMCA are rarely involved in atypical MI cases.

### Distribution of echocardiography results with typical and atypical MI

**Figure 3:**
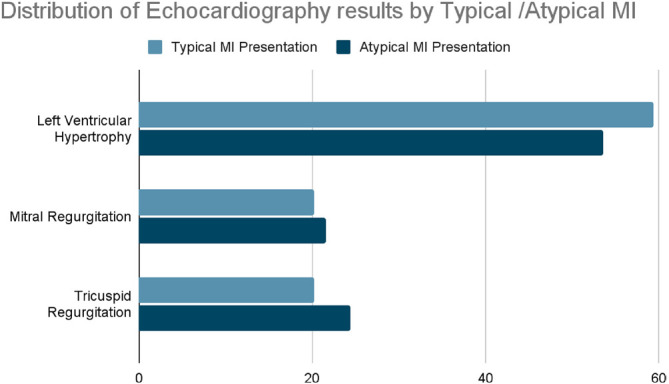
Distribution of echocardiography results by typical/atypical MI in the study sample (Amman, 2024). Left ventricular hypertrophy was the most common echocardiography finding in both groups (typical MI: 59.4%, atypical MI: 53.61%), followed by tricuspid regurgitation (20.3% and 24.74%, respectively).

Figure 3 presents the distribution of echocardiography results among patients with typical and atypical MI. The highest percentage of echocardiography results in both groups was left ventricular hypertrophy, with 59.4% of typical MI cases and 53.61% of atypical MI cases. Following this, tricuspid regurgitation was observed, accounting for 20.3% of typical MI cases and 24.74% of atypical MI cases.

## Discussion

In our study of 267 MI cases, 60.3% were typical and 39.7% atypical. Both types were most common in patients aged 64 or older, with no significant age association. Males predominated in both groups, and there was no significant sex association. Atypical MI cases had a higher prevalence of a history of coronary artery disease, 38.7%, and diabetes 66.0% compared to typical cases. Hypertension was common in both MI types, while hyperlipidemia was less prevalent with no significant association. Smoking history showed no significant differences. These findings are consistent with some studies, while others report differing results. While our study did not find any considerable age association, Ochiai and Beatrice documented that elderly patients (over 85) frequently present with atypical or absent myocardial ischemia symptoms, complicating diagnosis. They observed that many elderly patients with Q waves on ECG do not report precordial pain, in contrast to younger patients (under 65).^9,10^ In previous studies, the association between sex and MI presentations was debated, with findings varying and no consensus reached. While our study found no significant association, most studies support the finding that women are more likely to experience atypical symptoms. DeVon suggested that these atypical symptoms may lead to delays in diagnosis and treatment, resulting in poorer outcomes for women compared to men.^4^ Brush supported this view, noting that the difference might explain the higher missed diagnosis rate in young women with AMI.^11^ Joseph et al. emphasized the need for a gender-specific approach to prevent delays in diagnosis and care for women.^12^ However, Ferry contradicted these views, indicating that typical symptoms are more prevalent and have greater predictive value in women than in men with MI.^13^ Omran found in Jordan that women reported additional symptoms such as general weakness, sweating, nausea, and fatigue, with greater general weakness and sweating compared to men.^8^ Schulte added that females are likely to experience prodromal symptoms such as fatigue in the days preceding an MI.^14^

The demographic and cultural context of our Jordanian study may affect the findings related to age group and sex. Factors such as local healthcare practices, dietary habits, physical activity, and prevalent health conditions could influence MI presentations differently than in other regions. Additionally, cultural attitudes toward health and medical care may impact symptom reporting and healthcare utilization. For example, there is a prevalent belief among Jordanian women and elderly individuals that medical symptoms will resolve on their own with rest and patience rather than requiring immediate medical intervention. Furthermore, genetic factors unique to the Jordanian population could play a role in the variability of MI presentations and associated comorbidities. This context-specific influence highlights the importance of considering regional differences when interpreting clinical data and developing targeted interventions.

On the other hand, many studies support our finding that atypical MI cases exhibit a higher prevalence of a history of coronary artery disease 38.7% and diabetes 66.0% compared to typical cases. Hypertension was common in both MI types, while hyperlipidemia was less prevalent with no significant association. However, the association with diabetes, in particular, has been extensively highlighted in the literature and is emphasized more than any other comorbidity in the context of atypical MI. Shem-Tov’s analysis of 314 articles on atypical MI in diabetic patients revealed that the prevalence of silent or atypical myocardial ischemia (SMI) can be as high as 60% when ischemia is present without visible infarction on ECG, especially with advanced imaging techniques like single-photon emission computed tomography. This study underscores the importance of recognizing predictive factors such as advanced age, diabetes, obesity, and dyslipidemia in assessing MI risk in diabetic individuals, attributing the higher incidence of SMI to neuronal damage affecting cardiac nerve supply.^15^ Halushko found that 91.67% of diabetic patients aged 62–86 experienced painless MI.^16^ Stacey discovered that even impaired fasting glucose levels are associated with unrecognized MIs after adjusting for multiple risk factors in a multiethnic population.^17^ Xiao’s analysis of 24,732 individuals revealed that higher longitudinal fasting glucose levels and greater intra-individual variability in fasting glucose over time are associated with an increased risk of SMI in a dose-response manner, underscoring the need for routine cardiac screening in individuals with elevated fasting glucose levels despite diabetes.^18^ Several comorbidities are associated with atypical MI presentations. These include common conditions such as hypertension (HTN), diabetes mellitus (DM), and dyslipidemia. Additionally, less typical comorbidities were noted, including recurrent reflux esophagitis, gastric villous adenoma, thrombotic thrombocytopenic purpura, post-surgery complications, asthma, polycystic ovarian syndrome, Reiter’s syndrome, prostate cancer, myeloproliferative neoplasm, and rheumatoid arthritis. DM, HTN, and dyslipidemia emerged as the most frequently reported and relevant comorbidities in these studies.^12^ Khan confirmed that prodromal symptoms were often present, with most patients exhibiting two to three of the four common comorbidities: DM, HTN, dyslipidemia, and substance abuse.^18^ Ochiai further contributed that comorbidities such as fibromyalgia, depression, Alzheimer’s disease, and vascular dementia can modify the clinical presentation of myocardial ischemia as these neuropsychiatric conditions have the potential to interfere with the painful sensation; thus, it can obscure the perception of chest pain, resulting in atypical symptom presentations. Moreover, cognitive impairments linked to conditions like heart failure and previous myocardial revascularization can additionally complicate the description of chest pain symptoms in elderly individuals with myocardial ischemia.^9^ Another theory that can explain the atypical presentation of MI is the convergence-projection theory, which suggests that when the central nervous system encounters overlapping sensory inputs from different body parts, it may struggle to differentiate them, leading to confusion and misinterpretation that manifests as referred pain. This phenomenon aligns with Scherer’s componential model of emotion, which considers emotions as processes involving interacting components like cognitive appraisal, physiological responses, and expression. This model supports the idea that complex, multicomponent processes, including sensory pathways, might overlap, resulting in “blended” or ambiguous interpretations of stimuli, as seen in atypical MI presentations with jaw, neck, abdominal pain, or fatigue rather than classic chest pain.^19^

We have studied and found that recurrence rates for MI were similar between typical 4.3% and atypical 5.7% cases, with no significant difference in the timing of recurrence. The LAD artery was the most commonly involved in both types of MI, with slightly higher involvement in atypical cases. Echocardiography showed left ventricular hypertrophy as the most prevalent finding in both groups, followed by tricuspid regurgitation. We aimed to investigate the distribution patterns of artery involvement and echocardiographic findings to explore potential variability between typical and atypical MI presentations. Despite observing differences in the participation of specific arteries and echocardiographic findings, statistical analysis did not reveal significant differences between the two presentation types. Additionally, we examined recurrence rates to assess their association with presentation type and prognosis but found no significant correlation, suggesting that the presentation type may not strongly influence prognosis.

Our study identifies key areas for clinical practice and future research. The link between atypical MI, coronary artery disease, and higher diabetes prevalence highlights the need for increased awareness and targeted evaluation in these patients. While no significant age or sex differences were found, clinicians should prioritize individual risk factors over demographic profiles. The similar recurrence rates and artery involvement across MI types suggest that recurrence risk is consistent regardless of presentation type. Additionally, the various echocardiographic findings emphasize the need for thorough monitoring in both typical and atypical MI patients. This study provides insights by analyzing symptoms, age, gender, comorbidities, recurrence rates, arterial involvement, and echocardiographic findings in both atypical and typical MI cases. This comprehensive approach contributes to a deeper understanding of the disease, ultimately improving diagnostic accuracy and patient care.

One of the limitations was the reliance on patient-reported symptoms, which introduces the potential for bias, such as underreporting or misinterpretation of symptoms. A future direction for research is to explore the longitudinal outcomes of atypical MI to gain a better understanding of its progression over time. Investigating the impact of atypical MI on long-term cardiovascular health and comparing outcomes across diverse populations could provide further insights. Additionally, focusing on the effectiveness of various diagnostic approaches and treatment strategies may improve early detection and management. Expanding research to include larger, multicenter cohorts would also enhance the generalizability of findings.

## Conclusion

Atypical MI made up 39.7% of cases. Compared to typical MI, atypical cases showed higher rates of diabetes and coronary artery disease. No significant differences were found in age, sex, or recurrence rates. Both types had similar artery involvement, with atypical MI showing slightly higher LAD artery involvement. Echocardiography revealed similar left ventricular hypertrophy in both, with tricuspid regurgitation more common in atypical MI. These results emphasize the need for better risk assessment and early diagnosis of atypical MI.

## Conflicts of interest

None.

## Figures and Tables

**Table 1.** Distribution of study sample by typical and atypical MI presentation (Amman, 2024).

	**Typical MI (*n* = 161)**		**Atypical MI (*n* = 106)**	
	**No.**	**%**	**No.**	**%**	***X* ^2^**
**Age group**					
Age ≤64	122	75.8	80	75.5	0.58
65−	30	18.6	22	20.8	
75+	9	5.6	4	3.8	
**Sex**					
Male	128	79.5	78	73.6	1.27
Female	33	20.5	28	26.4	
**History of coronary artery disease**					
No	124	77.0	65	61.3	7.62*
Yes	37	23.0	41	38.7	
**Smoking**					
No	62	38.5	61	57.5	9.32
Yes	99	61.5	45	42.5	
**Diabetes**					
Normal	88	54.7	36	34.0	11.1*
Diabetic	73	45.3	70	66.0	
**Hypertension**					
Normotensive	58	36.0	33	31.1	0.68
Hypertensive	103	64.0	73	68.9	
**Hyperlipidemia**					
Absent	130	80.7	82	77.4	0.44
Present	31	19.3	24	22.6	

*p < 0.05. Table 1 presents the distribution of the study sample by typical and atypical MI presentation. The majority of cases in both groups were aged ≤ 64 years (typical MI: 75.8%, atypical MI: 75.5%), while the 75+ age group was the least represented (5.6% and 3.8%, respectively). Males predominated in both groups (79.5% and 73.6%). A high proportion of patients had no previous history of coronary artery disease (77.0% and 61.3%). Smoking was more common in the typical MI group (61.5%), whereas nonsmokers were more prevalent in the atypical MI group (57.5%). Normal blood glucose levels were prominent in typical MI cases (54.7%), while diabetes was more common in atypical MI (66.0%). Hypertension was prevalent in both groups (64.0% and 68.9%). Hyperlipidemia was largely absent in both groups (80.7% and 77.4%). Significant associations were found with a history of coronary artery disease and diabetes (*X*^2^ = 7.62 and 11.1, respectively).

**Table 2.** Distribution of typical and atypical MI by manifestations (Amman, 2024).

	**Typical MI (*n* = 161)**		**Atypical MI (*n* = 106)**		
	**No.**	%	**No.**	%	***X* ^2^**
**Recurrent MI**					
No	154	95.7	100	94.3	0.41
Yes	7	4.3	6	5.7	
**Recent MI since**	**(*n* = 17)**		**(*n* = 24)**		
1–2 months ago	1	5.9	5	20.8	2.12
3 months ago	16	94.1	19	79.2	
**Hemoglobin on admission**					
7 gm – <11 gm	8	5.0	11	10.4	3.93
11 gm – <15 gm	103	64.0	70	66.0	
≥15 gm	50	31.1	25	23.6	
**Number of vessels involved**					
Free	39	24.2	63	59.4	34.1
1 vessel	68	42.2	27	25.5	
≥2 vessels	54	33.5	16	15.1	

*p < 0.05. Table 2 shows the distribution of MI manifestations in typical and atypical MI groups. The majority of patients in both groups had no recurrent MI (typical MI: 95.7%, atypical MI: 94.3%). Most recent MI cases occurred 3 months prior (94.1% and 79.2%, respectively). Hemoglobin levels between 11 and 15 gm on admission were common in both groups (64.0% and 66.0%). In the typical MI group, one vessel involvement was most frequent (42.2%), followed by two vessels (33.5%), while in the atypical MI group, no vessel involvement was most common (59.4%), followed by one vessel (25.5%). Creatinine levels below 245 mg/dL were prevalent in both groups (100.0% and 98.1%).
